# Characterizing the Structure and Interactions of Model Lipid Membranes Using Electrophysiology

**DOI:** 10.3390/membranes11050319

**Published:** 2021-04-27

**Authors:** Joyce El-Beyrouthy, Eric Freeman

**Affiliations:** School of Environmental, Civil, Agricultural and Mechanical Engineering, College of Engineering, University of Georgia, Athens, GA 30602, USA; joyce.elbeyrouthy@uga.edu

**Keywords:** model membranes, electrophysiology, membrane-particle interactions, lipid bilayer electrostatics, conductive channels, soft capacitor, impedance analysis

## Abstract

The cell membrane is a protective barrier whose configuration determines the exchange both between intracellular and extracellular regions and within the cell itself. Consequently, characterizing membrane properties and interactions is essential for advancements in topics such as limiting nanoparticle cytotoxicity. Characterization is often accomplished by recreating model membranes that approximate the structure of cellular membranes in a controlled environment, formed using self-assembly principles. The selected method for membrane creation influences the properties of the membrane assembly, including their response to electric fields used for characterizing transmembrane exchanges. When these self-assembled model membranes are combined with electrophysiology, it is possible to exploit their non-physiological mechanics to enable additional measurements of membrane interactions and phenomena. This review describes several common model membranes including liposomes, pore-spanning membranes, solid supported membranes, and emulsion-based membranes, emphasizing their varying structure due to the selected mode of production. Next, electrophysiology techniques that exploit these structures are discussed, including conductance measurements, electrowetting and electrocompression analysis, and electroimpedance spectroscopy. The focus of this review is linking each membrane assembly technique to the properties of the resulting membrane, discussing how these properties enable alternative electrophysiological approaches to measuring membrane characteristics and interactions.

## 1. Introduction

Cell membranes are semi-permeable barriers surrounding cellular organisms, separating the intracellular components from the extracellular environment [1]. Since cell membranes provide the cellular architecture enabling the distinction between adjacent regions of the cell and intracellular compartmentalization, these lipid barriers are a fundamental scaffold for inter and intracellular communication and exchange [2]. Within individual cells, membranes permit for multiple molecular reactions to occur simultaneously via membrane compartmentalization [3,4]. Within the membrane itself, multiple lipid domains coexist in different parts of the membrane, controlling various cellular activities [3]. These lipid domains undergo changes in phase separation and overall lipids packing essential to cell differentiation and proliferation [5]. Furthermore, membrane electrochemical properties are vital for the cell’s functionality, such as the propagation of action potentials and maintaining intracellular compositions [6]. Since the membrane acts as a differentiating limit between the cytosol and the extracellular environment, it also governs cell-to-cell communication [2,5]. External stimuli are detected by the membrane’s corresponding receptors initiating complex molecular reactions through ion channels opening or closing depending on the reaction launched [7,8]. Thus, the membrane is a key element in the life of individual cells as well as in tissue maintenance. Investigating its properties and dynamic behavior opens doors for advancements in pharmaceuticals [9,10], synthetic biology [11,12], and bioinspired materials [13,14].

**Figure 1:**
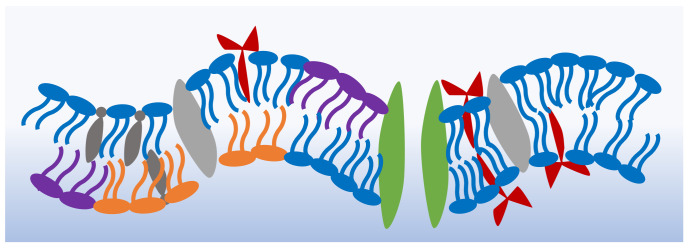
A schematic illustrating the complexity of natural cell membranes. The base structure of these barriers is a double layer of phospholipids. Transport proteins, sterols and other biomolecules are present in different parts of the membrane depending on the cell’s role and life cycle stage.

The cell membrane’s primary structure is a double layer of phospholipids that holds within its leaflets varying components including the proteins, peptides and sterols necessary for its functionality [1]. Figure 1 shows a schematic of the shape and possible structure of a generic cell membrane [15,16,17,18]. Each membrane or region within the membrane possesses a particular molecular combination that produces varying bulk properties. For example, drug-resistant cancer cells show a higher membrane bending rigidity in comparison to drug-sensitive cells [19,20], and the negatively charged exoplasmic surface of gram-negative bacteria makes them an easier target for cationic antibacterial peptides [21,22]. The two lipid leaflets forming the cellular envelope exhibit varying compositions. For example, cholesterol is more abundantly found in the inner leaflets of plasma membranes [23], whereas membranous domains, or lipid rafts, are mainly formed in the exoplasmic leaflets [24]. Cellular membranes rely on their asymmetry for stability, shape, permeability as well as membrane potential activities. The membrane asymmetry presents a constant state of non-equilibrium which is maintained by continuous active processes [25]. 

Phospholipids are amphiphilic molecules possessing a hydrophilic headgroup and two hydrophobic fatty acid chains [26]. This amphiphilic structure enables their self-assembly whenever dispersed in a polar-apolar medium [27]. The middle layer of the cell membrane consists of the fatty acid chains bonding through hydrophobic forces, whereas the two outer layers are primarily the hydrophilic head groups. Since the membrane’s hydrophobic interior is near-impermeable to dissolved species within the aqueous phases, transmembrane exchange is primarily handled through embedded channels, and transport proteins contained within the membrane interior [1]. Furthermore, this middle layer provides an electric permittivity that is substantially lower than that of the outer hydrophilic regions leading to the traditional membrane’s electrical representation: a capacitor in parallel with a resistor [28]. This analogy is the basis of electrical investigations of membrane structure, dynamics and nanoparticle interactions.

One technique for characterizing the electrical properties of the cell is the patch-clamp technique [29], where an electrode-pipette comes in direct contact with an isolated cell bathed into an electrolyte solution mimicking its physiological environment [30,31]. Silver-silver/chloride (Ag/Ag-Cl) electrodes connected to a patch clamp amplifier allow for either a clamped voltage or current, measuring the membrane response and producing current-voltage relationships for further study. The produced electric field here falls primarily across the membrane interior through the separation of charged ions, mimicking membrane potentials within the body. Individual patches of the membrane can be isolated by adjusting the position of the electrode. However, this technique requires precise positioning and presents unique challenges due to the complex and delicate structure of the cells. In addition, studying the intertwined membrane components within a single patch or entity of a complex natural membrane often prevents the ready isolation of the desired agent-membrane interactions. Thus, to elucidate the components of a certain mechanism, one needs to recreate the lipid membrane in a more controlled laboratory environment, commonly achieved through creating synthetic, or model, membranes. Model membranes are synthetic double layers of phospholipids mimicking the core structure of the biological membrane. They present a tailorable model platform for simulating cellular environments and allow for a better control over simulated external conditions.

In the last decade, multiple reviews discussing different model membrane formation techniques combined with electrophysiological characterizations have been published. Siontorou et al. presented the advancements in model membrane platforms, suspended and supported, while focusing on their applications in biosensing and characterization [32]. Khan et al. described various membrane-protein mechanics based on electroimpedance studies for solid supported model membranes and pore-spanning membranes [33]. Similarly, Grewer et al. compared protein transport in artificial lipid membranes to natural cell membranes focusing on the patch-clamp technique [30]. Nanomaterials and nanoparticles interactions at the membrane level were also discussed by Wu and Jiang [34], and Rascol et al. [35], respectively, while not limiting the characterization techniques to electrophysiological approaches. The review presented here instead focuses on some of the most relevant and recent electrophysiological approaches for investigating membrane structure and interactions, highlighting in particular how the selected method for membrane formation influences the available methods for characterization.

First, four of the most common model membranes are presented along with their properties and experimental artefacts resulting from their mode of production. Section 2.1 discusses lipid vesicles or liposomes formed in aqueous solutions or microfluidic channels. Section 2.2 presents pore-spanning membranes formed at the orifice of a hydrophobic wall. In Section 2.3, solid supported membranes formed at the surface of a hydrophilic support are explained. In Section 2.4, two droplet-based membranes–droplet on hydrogel bilayer and droplet interface bilayer–are described highlighting their emulsion properties. Next, three major concepts of membrane electrophysiology are explained along with the membrane characteristics they underline. Section 3.1 discusses membrane conductance measurements, highlighting the mechanics of transmembrane exchange. In Section 3.2, the membrane response to a varying electric field–electrowetting and electrocompression–is presented based on changes in membrane capacitance or membrane generated current. In Section 3.3, electroimpedance spectroscopy is explained, which interprets changes in membrane properties using impedance models. Throughout this discussion, we focus on linking the electrophysiology approaches to the model membrane properties and constraints, while highlighting how the membrane structure and interactions may be assessed.

## 2. Model Membranes: Manufactures and Resulting Properties 

Model membranes reproduce the fundamental structure of cellular membranes: a double layer of phospholipids. Each model membrane platform is unique and leads to a different environment for studying membrane structures and interactions. This section presents four of the most common model membranes, explains their formation process, and discusses their resulting properties and how these properties may influence measurements of membranous phenomena.

### 2.1. Liposomes 

**Figure 2:**
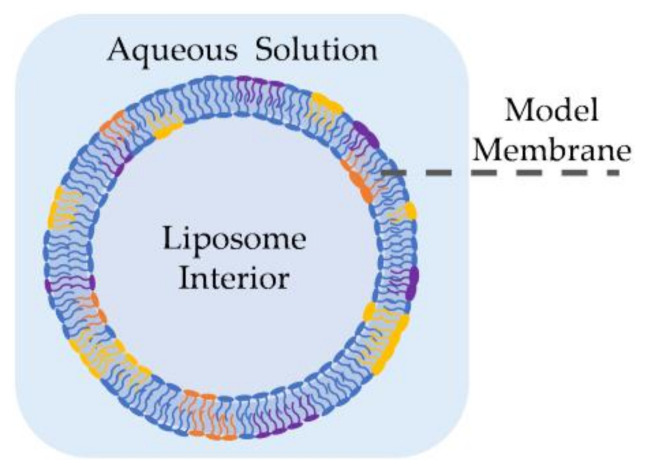
A cross-section of a liposome, or a lipid vesicle. Liposomes are model membranes recreating a lipid bilayer, while resembling cells in their shape and size—Especially through giant unilamellar vesicles. Liposomes can be formed through electroformation, phase transfer or microfluidic jets.

Liposomes, also called lipid vesicles, are one of the earliest forms of synthetic membranes [36]. As shown in Figure 2, they are spherical lipid bilayers formed in an aqueous environment, commonly through electroformation [37,38,39], phase transfer [40,41,42] or microfluidic jets [43,44]. Liposomes may be formed in different sizes and distributions [45], but giant unilamellar vesicles (GUVs) are often employed as they are comparable in size and shape to living cells [44]. GUVs may be formed using electroformation [37], where a volatile solvent, such as chloroform or methanol, containing the desired lipids is placed on a conductive surface–commonly an indium tin oxide (ITO) slide–followed by an overnight evaporation to form dry lipid films. These dry films are then rehydrated with sucrose solution and the vesicles are formed by applying an AC voltage across the conductive surface, where the voltage frequency and amplitude are tuned to reach the desired liposome size. In phase transfer [40], an aqueous droplet, submerged in a lipid-dispersed oil medium is coated with these lipids through their amphiphilic-driven self-assembly. The coated droplet is then added onto a separate water-oil lipid monolayer. The difference in salt concentrations between the water droplet and the secondary aqueous solution drives the droplet into the planar lipid sheet forming a spherical double layer or, a liposome. Elani et al. showed that this approach enables the formation of adjacent compartments mimicking compartmentalization observed in living cells [46]. Furthermore, they successfully formed and mechanically investigated asymmetric liposomes [47] as well as thermally controllable lipid vesicles [48]. Authors noted residual solvent in between the leaflets when created using phase transfer. However, this was not an issue in the microfluidic jet technique, where a focused fluid flow is applied to a planar bilayer formed at a water-oil-water interface, generating multiple lipid vesicles [43,44].

Liposomes are commonly used to investigate membrane permeability through fluorescence [49] or radioactive tracking [50], in addition to permitting measurement of some mechanical properties [47] such as membrane bending rigidity [51,52]. The shape of liposomes resembles that of natural cell membranes in providing a closed, continuous membranous shell around their contents. This renders them a reliable platform for the study of nanoparticles-membrane interactions [53,54,55], especially nanoparticle uptake [56]. In addition, these lipid vesicles form the basic structure of multiple drug-delivery nanocarriers [9,57,58]. Encapsulating a certain drug, usually of a toxic or fragile nature, inside a closed membrane allows for its safe transport across the organism until it reaches its target destination [59,60]. The transport and delivery of the drug is more effective, better controlled and safer through lipid-composition alternations and surface manipulations [61,62,63]. For example, thermosensitive liposomes, formed by mixture of low-temperature sensitive phospholipids, enable the localized release of toxins in the diseased area through temperature manipulation [64].

Liposomes are geometrically comparable to natural membranes allowing for studies of membrane mechanics [65,66,67], undulations [52], and surface interactions [68]. Furthermore, single-channel recordings of transmembrane activity in liposomes is possible by means of the patch-clamp technique [68]. However, since many electrical methods for liposome characterization involve placing the liposomes between two electrodes and supplying an external field rather than a localized field directly across the membrane itself, liposomes electrophysiological studies are primarily limited to single-channel patch-clamp measurements and are not a point of emphasis within the scope of this particular review article.

### 2.2. Pore-Spanning Membranes

**Figure 3:**
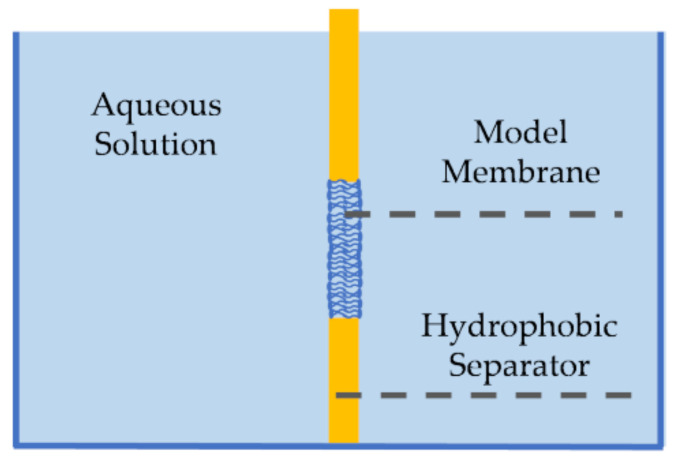
Pore-spanning membranes are planar lipid bilayers formed at the orifice of a hydrophobic separator between two aqueous solutions. The membrane can be achieved through the painting or folding approach. In the painting method, lipid-dispersed solvent is placed in the separator hole by painting it with a syringe or a brush. The bilayer is then formed through lipids self-orientation. In the folding method, the lipid monolayers are initially formed at the water-air interface while the orifice is higher than the water level. Then, pulling the hydrophobic separator downwards, the monolayers follow through hydrophobic bonding and the bilayer is formed in the orifice.

First introduced by Mueller et al., pore-spanning membranes, also referred to as black lipid membranes, are formed at the opening of a hydrophobic separator (or septum) between two aqueous baths [69,70], as seen in Figure 3. These membranes were first created using the painting technique [69], where a membrane-forming solution would be spread across the orifice by means of a brush or a syringe. First, the solvent solution–commonly decane oil containing phospholipids–is brushed on both sides of the aperture. Due to the amphiphilic nature of the lipids, they self-assemble such that the hydrophilic heads are oriented towards the aqueous baths. Since the separator is hydrophobic, the solvent moves towards its surface, partially expelling itself from between the monolayers, forming the lipid membrane at the aperture between the two baths. Decane is often used as the solvent in this technique due to its high volatility and low viscosity compared to higher chain oils, enabling partial evaporation and easier relocation from between the monolayers, and thus a proper membrane formation [71]. Silver/silver-chloride (Ag/AgCl) electrodes are placed in the aqueous solutions on opposite sides of the membrane enabling electrophysiological measurements. Note that the painted membrane may contain excess residual solvent, as the short chain oil does not completely expel itself from between the lipid leaflets, leading to soft or highly elastic membranes [72,73]. The amount of residual solvent within the membrane has been reduced by various efforts including coating the aperture with an amphiphobic agent [74], decreasing the control temperature to below the oil freezing point [75], and using longer chain solvents that are unable to distribute within the membrane interior [72,76]. In addition, the formation of asymmetric membranes, where the two leaflets are composed of different lipid combinations, requires additional layers of formation [77]. 

One decade later, Montal and Mueller introduced the folding approach for creating pore-spanning membranes, by folding two air-water lipid monolayers into the hydrophobic orifice [70]. In this technique, two lipid monolayers are first formed at the water-air interface separated by the solid septum. The two monolayers are formed by adding phospholipids-dispersed volatile solvents, such as chloroform or ethanol, on the surface of the aqueous solutions. The solvents then evaporate leaving the dry film at the water-air interface. The hydrophobic separator orifice, which is originally higher than the monolayers level, is slowly pulled downwards dragging the two monolayers along and forming the bilayer through hydrophobic affinity. The rate of displacement of the separator should be slower than the rate of monolayers bonding to ensure a successful membrane formation. Since there is no initial solvent residue, the folded membrane is solvent-free and closer in thickness to that of living membranes. Additionally, asymmetric membranes can be directly formed through the folding technique by originally placing different phospholipids on the two aqueous surfaces [78,79].

Recent pore-spanning membrane platforms involve lipid bilayers supported over multiple pores [80], as well as thin-film pressure balances, which are combined with electrophysiology for precise characterization of large area model biomembranes (LAMBs). These systems have been presented by Beltramo et al., providing control over membrane tension with varying solvents [72,81] and demonstrating asymmetric membrane formation [82].

These modes for forming pore-spanning membranes lead to sealed and tightly-packed lipid bilayers with a high innate membrane impermeability, and thus a high electrical resistance [70,83]. This high innate resistance rendered these membranes as ideal for studies on transmembrane exchange [70,79,84,85]. Furthermore, the membrane area is geometrically constrained by the surrounding orifice limiting its ability to adjust in response to externally applied forces [69,70,71].

### 2.3. Solid Supported Membranes 

**Figure 4:**
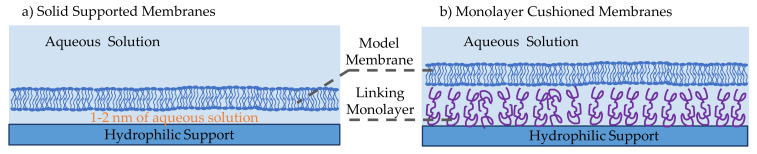
Model membranes are formed on a solid substrate in two configurations. (**a**) Solid supported membranes are formed at the planar surface of a hydrophilic solid support. The bilayer is formed through LB/LS method or vesicle fusion method or a combination of both. The resulting membrane is stable, mechanically robust and long-lasting as a result of localized tight lipids packing and the presence of the underneath solid support. (**b**) The introduction of a linking interstitial region between the solid support and the membrane leads to a larger aqueous environment beneath the membrane. This facilitates the introduction of proteins and larger biomolecules in a safe and unconstrained setting. The joining monolayer can be formed through a polymer, a protein, thiolipids or other amphiphilic molecules.

Solid supported membranes (SSMs) are model membranes that are formed on a hydrophilic solid support in an aqueous medium, as illustrated in Figure 4a [14,33]. These have shown to be more robust and stable than previously developed model membranes owning their stability to the localized tight lipids packing and the solid supporting scaffold. Their robustness and stability lead to their popularity in molecular electronic microfluidic chips [14,86,87]. They were first introduced by Tamm and McConnell, where two water-air monolayers were deposited on a hydrophilic solid support such as silicon, glass and quartz [88]. In this initial work, membranes were formed through the Langmuir-Blodgett and Langmuir Schaefer (LB/LS) technique, while others have later successfully formed these membranes through vesicle fusion or through a combination of both. In the LB/LS technique, the lipid monolayer is formed at a water-air interface through phospholipids self-assembly, then a hydrophilic aperture is displaced across while adhering the lipid sheets on its surface. The film is then placed horizontally on top of the other monolayer and pushed under the water level until deposited on the bottom of the reservoir. The second approach to forming SSMs is through vesicle fusion [89], where small unilamellar vesicles are formed and dispersed into the aqueous solution covering the hydrophilic substrate. Driven by hydrophilic favorability, the vesicles adsorb and unfold onto the hydrophilic surface, forming a planar lipid bilayer. The third approach is a combination of these two techniques, where the bottom lipids sheet is formed by means of the Langmuir-Blodgett monolayer and the top lipids sheet is formed through vesicles unfolding [90]. This combined approach is mostly used for the formation of asymmetric membranes–different lipids forming the two leaflets. The material of the hydrophilic support has been varied over the years, depending on the required membrane properties and the technique used to study the membrane. Commonly, silicon or mica are used in atomic force microscopy as they provide flat and smooth surfaces [91,92], gold and silver are adopted during surface plasmon resonance technique [93,94,95], silica and borosilicate glass are used in optical-based techniques [96,97], whereas Indium-Tin-Oxide (ITO) glass is the most suitable for electrophysiology studies due to its high electrical conductivity [98,99]. 

Solid supported membranes in their original form were not optimized for incorporating proteins and peptides. The resulting 1–2 nm aqueous layer between the membrane and the solid substrate [97,100] is insufficient for these large molecules to freely move, and in most cases, they are exposed to the solid surface leading to denaturation. Consequently, monolayer cushioned membranes were introduced [101]. As shown in Figure 4b, these membranes differ from previously discussed SSMs by the presence of an additional monolayer between the solid substrate and the membrane [102,103,104,105,106]. The intermediate amphiphilic layer substantially increases the aqueous layer thickness leading to the possibility of adding membrane-active molecules and observing their behavior in an unconstrained environment [107]. The biomolecule used should be amphiphilic, soft, able to attach to the membrane and the hydrophilic surface while minimally interactive with the studied proteins avoiding unwanted interactions [103]. Conventionally, differentiation occurs between polymer-cushioned membranes and tethered membranes. The most common polymers adopted are polyelectrolytes polymers [105,108,109], which are driven by electrostatic forces to abide to the solid surface, and lipopolymers [110], which are lipid-like polymers that bind themselves between the phospholipids and the solid substrate. Tethered bilayers lipid membranes, or tBLMs, are supported via tethering of thiolipids [111,112,113], which are amphiphilic molecules possessing a hydrophilic separator [114]. Zhang et al. used a conductive polymer–poly(3,4-ethylenedixoythiophene) polystyrene sulfonate (PEDOT:PSS)–as the membrane’s cushion forming a biological transistor [115]. In addition, the polymer layer can be altered to form a complex mesh similar to that of the extracellular matrix, improving the system’s physiological similarity [116]. 

The membrane resistance in this case is approximately one order of magnitude lower than that of cell membranes and several orders of magnitude lower compared to other model membranes [98,99]. This has been interpreted by the presence of scattered voids in between the lipids packing caused by the solid surface roughness [117]. Multiple successful efforts have been presented to minimize these membrane defects and increase its resistance, including the addition of a hydrogel layer leading to a smooth and functional surface for the compact attachment of tethered-protein, forming a tightly packed, defects-free giga-resistive membrane [112,118]. In a recent study, solid supported membranes were formed through polar lipid fraction E (PLFE) remained stable in a microfluidic chip for 50 h while maintaining a constant impedance value [119]. In the literature, electrophysiological studies of these membranes typically involve electroimpedance spectroscopy (EIS), which will be discussed in Section 3.3. The EIS works well with solid supported membranes as it characterizes the impedance of the individual layers. 

Another model membrane technique that can be described as solid-supported is called the “tip-dip” technique [120]. First introduced by Coronado and Latorre, the model membrane is formed at the tip of a few micrometers wide glass pipette. In a lipids-dispersed aqueous solution, the hydrophilic glass pipette is submerged, and a lipid monolayer is formed at the water-air interface surrounding the pipette. Once the monolayer is formed and stabilized the pipette is removed and reentered into the aqueous solution several times, hence the “tip-dip” term. This manipulation of the pipette ensures the formation of the lipid bilayer at its tip when submerged in the aqueous medium. The preference of this approach over previously discussed solid supported membranes is enhanced when interested in single-channel recordings [121,122]. The first attempt of these membranes led to 5–20 GΩ membranes while following efforts reached up to 100 GΩ by using a polyethylene glycol (PEG)–coated gold electrode [123]. In a comparison between membranes formed by the tip-dip method and membranes formed by the painting technique in studying gramicidin, Matsuno et al. found that even though both techniques enable reliable channel recordings, the tip-dip approach formed more stable and long lasting membranes allowing for minutes long recordings otherwise unachieved [121]. Furthermore, membranes formed at the tip of a glass electrode present the additional advantage of reversible membrane formation. Shoji et al., developed a gold-based electrode where lipids sheets were formed on gold-oil and water-oil interfaces and showed a directional dependency on protein gating [124], while Hirano et al., expanded this technique towards immobilizing proteins on hydrogel beads for prompt constitution of channels [125]. Challita et al. formed membranes at the interface of an aqueous droplet and a polyethylene glycol dimethacrylate (PEGDMA) hydrogel pipette, submerged in an oil dish [126] and emphasized reliable and repeatable membrane formation. In this work, membranes were formed by piercing a lipid-oil medium with a lipid-coated electrode to contact another lipid-coated aqueous droplet.

### 2.4. Membranes Formed at The Interface of Immiscible Fluids 

In this section, two emulsion-based model membrane techniques will be presented, where at least one of the lipid monolayers is formed at the surface of an aqueous droplet submerged in an oil medium. The immiscibility of water droplets in oil drives the formation of lipid sheets at the hydrophilic-hydrophobic interface. These microfluidic-based model membranes allow for the utilization of emulsion science to determine membrane mechanics. 

#### 2.4.1. Droplet on Hydrogel Bilayers

**Figure 5:**
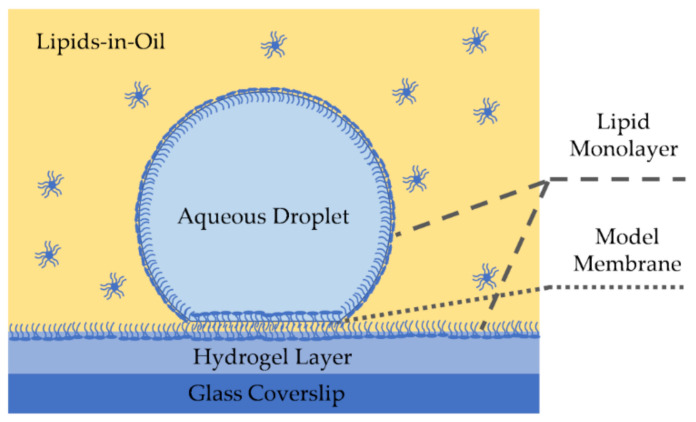
Side view of a droplet on hydrogel bilayer, or a membrane formed at a water-hydrogel interface in an oil medium. Here a hydrogel layer and a water droplet are submerged in a lipid-dispersed oil solution and the amphiphilic molecules self-assemble at the oil-hydrogel and at the oil-water interfaces forming monolayers. Placing the aqueous droplet on the hydrogel surface enables membrane formation.

Emulsion-based lipid membranes have been reported since 1966 by Tsofina et al. and others [127,128,129]. However, it was not until the early 2000s that these techniques gained popularity for electrophysiological studies. Droplet on hydrogel bilayer, or a DHB, forms a model membrane at the interface of a water droplet and a hydrogel surface submerged in an oil medium [73,130,131], as shown in Figure 5. Molten [73], or spin-coated [132], hydrogel is placed on a glass coverslip forming a hydrophilic surface at the bottom of an oil well. The desired phospholipids are dissolved in the oil medium–or in the oil and droplet–and due to their amphiphilic property, self-assemble at the hydrogel-oil interface forming a lipid monolayer. Submerging a nanoliter aqueous droplet in the reservoir forms the second monolayer at the water-oil interface surrounding the droplet’s surface. Once the monolayers are formed and stabilized, the droplet is placed on the hydrophilic surface leading to the formation of the lipid membrane at that interface, and electrophysiology measurements are enabled through the presence of Ag/Ag-Cl electrodes on either side of the membrane [73,131].

DHBs have shown to provide high lateral lipid mobility as the smooth and homogeneous underlying hydrogel layer minimizes area defects leading to a high innate resistance [131]. Lateral lipid mobility enhances these membranes biological relevance and makes them a strong candidate for membrane-particle diffusion studies [130]. Additionally, the DHB setup enables full visualization of the membrane surface area within the focal plane of an inverted microscope [73], leading to an advantage in measuring properties dependent on the membrane area such as specific capacitance.

#### 2.4.2. Droplet Interface Bilayers

**Figure 6:**
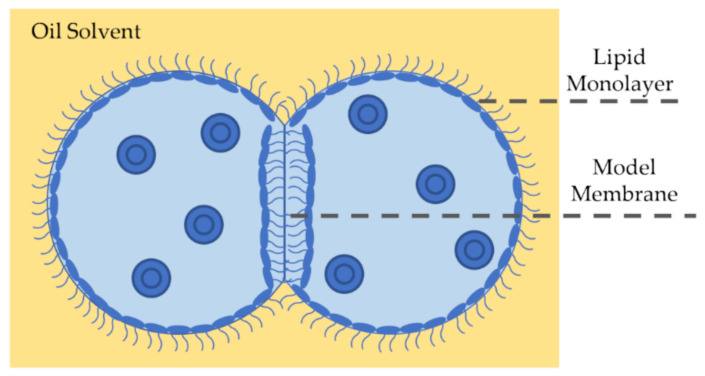
A DIB is a model membrane formed at the interface of two lipid-coated droplets in oil. Submerging lipid-dispersed aqueous droplets in an oil medium leads to the self-assembly of the amphiphilic molecules at the water-oil interface forming the monolayers. Note that it is possible to have the phospholipids dispersed in oil or water. Placing the droplets into contact, the membrane spontaneously forms at their adhered interface. The droplets contact angle links the membrane mechanics to the DIB’s equilibrium state is dictated by the balance of surface tensions.

Droplet interface bilayers, or DIBs, are model membranes formed at the adhered interface of two aqueous droplets submerged in an oil medium [133,134,135], as seen in Figure 6. Similarly to DHBs, DIBs require the presence of two immiscible fluids, aqueous droplets in oil. Lipids can be dispersed in the aqueous medium or in the oil medium, or both. Due to their amphiphilic nature, the lipids self-assemble around the droplets surfaces and form lipid monolayers at the water-oil interface, which are then brought together forming the membrane. The droplets are often suspended from Ag/Ag-Cl electrodes enabling electrophysiological studies. In the case of droplet-based membranes, the equilibrium position of the droplets and the equilibrium membrane capacitance are denoted by the surface tension balances.

The DIB technique enables the formation of freestanding liquid-in-liquid membranes able to respond to externally applied forces, including the electric field across the membrane [136,137]. Note that despite the droplets’ attachment to silver electrodes, these wires are micrometers thick in diameter leading to a minute physical constraint, which does not overly restrict the DIB interface from expanding or shrinking as needed. Thus, the DIB allows for a direct link between interfacial tensions and membrane biophysics, permitting visual measurements of membrane qualities. Membrane surface tension is measured by balancing forces at the triple point of contact. The system’s equilibrium is defined by the contact angle between the droplets and it is utilized to monitor the behavior of membrane tension under changing conditions such as a varying electrical field [137]. DIBs present another advantage as they form asymmetric membranes in a simple yet controllable manner by dispersing different lipid mixtures in each droplet [138,139]. Furthermore, these emulsion systems allow for the assembly of membranous networks for investigating synthetic tissues [140] and bespoke model environment for studying transmembrane exchanges [141]. 

DIBs present challenges including the assumption of spherical droplets and complications arising from the surrounding oil reservoir. When investigating DIB mechanics and observing droplets through an inverted microscope, it is often assumed that the droplets are spherical, and that the membrane surface area is consequently circular. However, the presence of surfactants at the droplets surfaces leads to a reduction in the water-oil surface tension [142] to magnitudes of approximately 1 mN/m [136,137,143], making the droplets sag from perfect spheres to hanging droplets, and thus the membrane area is an ellipse rather than a perfect circle. This issue has been addressed by multiplying the membrane area by a compensating factor depending on the monolayer surface tension and the oil density [137], or by placing a side view camera allowing for measurements of both principal diameters of the elliptical membrane [136,143]. As for the immense oil reservoir surrounding these membranes, it largely influences the resulting thickness and elasticity of the produced membranes [73]. The solvent’s viscosity affects the intensity and the pace at which the membrane responds to external stimuli, by inducing a resistance that the leaflets must overcome to adjust the membrane’s geometry accordingly. Moreover, when amphiphilic molecules are dispersed within the droplets, the encapsulating oil-water interface is likely to attract the molecule and drive it from its desired location–in between the membrane leaflets which may be measured through electrophysiological techniques–to its more favorable hydrophobic environment, interfering with the designed experimental conditions. 

## 3. Electrophysiological Methods for Characterizing Lipid Membranes 

Electrophysiology is a fundamental technique in cellular biology, especially for studying cell membranes. Ag/Ag-Cl electrodes are introduced to the aqueous phases adjacent to the membrane and connected to a patch-clamp amplifier. Voltage-clamp is the primary method discussed here, where the voltage drop between a source electrode and the ground is clamped to a desired waveform function, and the current necessary to maintain that voltage is recorded. The Ag/Ag-Cl electrodes ensure that this voltage drop falls primarily across the lipid membrane, and measurements are typically conducted in properly-grounded low-noise environments, enabling measurements of the current within the picoamps range. Precise current-voltage relations are produced for lipid membranes through this approach and translated into membrane properties and interactions through various interpretations of the membrane structure. 

In this review, electrophysiology-based characterization approaches are presented along with the membrane properties they assess. While multiple model membrane properties are mentioned herein, focus will be placed on four membrane-defining characteristics: membrane capacitance *C_s_*, membrane conductance *G_s_*, membrane intrinsic potential ∆*φ*, and membrane surface tension *γ_b_*. These aspects are used to reveal membrane structure and changes in their values will be interpreted into membrane dynamics. 

**Figure 7:**
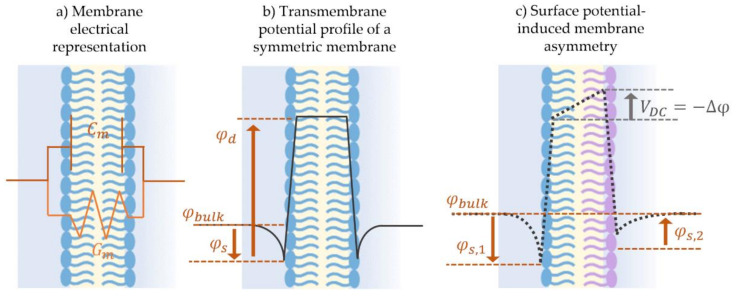
Membrane electrostatics. (**a**) Membranes are electrically modeled as a capacitor and a resistor in parallel. Membrane capacitance arises from the permittivity difference between the inner hydrophobic layer and the two outer hydrophilic surfaces, providing the membrane its ability to retain charge. Membrane resistance arises from its impermeability to dissolved species, except in the presence of conductive channels or pores. (**b**) The monolayer surface potential, *φ_s_*, and dipole potential, *φ_d_*, summarize the overall transmembrane potential profile. In the case of a symmetric membrane, lipid leaflets are identically composed and consequently show similar surface and dipole potentials. This leads to a symmetric transmembrane potential profile, as indicated by the solid lines. (**c**) In the case of an asymmetric membrane, the lipid leaflets are formed with different lipid mixtures leading to different surface and/or dipole potentials. This schematic illustrates an example of one leaflet possessing a lower surface potential generating a mismatch across the membrane, characterized by the asymmetric potential in the bulk, *Δφ*. When electrodes are placed in the bulk and the membrane is short-circuited in voltage-clamp mode at 0 mV, an electric field is produced across the membrane as the asymmetric values in the bulk are corrected.

Model membranes are typically studied using voltage-clamp mode, where a voltage is prescribed and the current necessary to maintain it is measured. The applied voltage and the generated current are then interpreted using an electrical model of the membrane to separate contributions from its capacitance and conductance. As shown in Figure 7a, the standard electrical representation of membranes is a variable capacitor in parallel with a resistor [144,145]. The current *I(t)* passing across the double layer possesses a capacitive and a resistive component as following:

(1) $$I\left(t\right)=C_{m}\frac{dV\left(t\right)}{dt}+V\frac{dC\left(t\right)}{dt}+G_{m}V\left(t\right)$$

where *V(t)* is the voltage across the membrane, and *C_m_* and *G_m_* are the membrane total capacitance and conductance, respectively. The first two terms on the right-hand side of the equation denote the capacitive current taking into consideration the soft nature of this biological capacitor. The third term represents the resistive current and is calculated solely through the direct voltage component. Varying the nature of the applied voltage allows for the isolation of the membrane electrical properties, *C_m_* and *G_m_*, which may be then used to infer membrane structural qualities. It is important to note that these are the properties of the membrane as a whole.

Membranes owe their capacitive nature to the dielectric permittivity difference between the hydrophobic fatty acid chains$—\varepsilon_{r}\;\sim \;2.2$ [146]—forming the membrane’s middle layer, and the hydrophilic headgroups forming the two outer surfaces$—\varepsilon_{r}\;\sim \;5$ [146]. This difference in permittivity leads to a parallel plate capacitor-like structure and behavior, where the capacitor’s permittivity is approximated as that of the hydrocarbon interior [147]. Model membrane specific capacitance, *C_s_* denoted as capacitance per unit area, depends on the lipids and solvent used, the bilayer’s geometry, as well as the forces applied on the fluidic system [148]. It is used to calculate membrane dielectric thickness according to the parallel plate capacitor equation. Note that this membrane thickness is the water-to-water distance across the phospholipids double layer, which is sometimes altered by the presence of water molecules near the hydrophobic group due to dynamic fluctuations [70,149]. The resistive component, *G_m_*, on the other hand, depends on the membrane’s permeability or the presence of ions conductive channels. The cell membrane’s main role is a selective barrier as it reacts to each pore-forming agent differently [150]. Defects, pores, and channels across the membrane increase the membrane’s conductance as ions travel through the pathways to the other side. A perfectly sealed membrane with no conductive channels, presents a high resistance in the order of giga-ohms and the electrical current is primarily capacitive. 

The approximation of the membrane as a capacitor and a resistor loses sight of its underlying electrochemical structure. The molecular composition of the individual lipid presents fixed charges along its profile producing localized electric fields. The position and amplitude of these fields establish the overall transmembrane potential profile across the membrane thickness [146]. In summary, each lipid leaflet possesses a surface and a dipole potential. First, the leaflet’s surface potential is induced by surface charge at the aqueous-phospholipid interface, and depends on the phospholipids charged headgroups as well as on the surrounding electrolyte concentration [151]. Second, the dipole potential is typically present at the linking group joining the hydrophilic and hydrophobic parts of the amphiphilic molecule [152], and this potential is largely independent of the aqueous solution and is a function of the selected lipids [153,154]. Any asymmetry between the leaflets concerning these underlying electrostatics generates a membrane potential, *Δφ,* characterized by the overall offset in the transmembrane potential profile. 

Not to be confused with the resting potential of natural membranes, model membrane potential discussed herein is the result of an imbalance between the leaflets electrostatics, induced by short-circuiting the model membrane through Ag/Ag-Cl electrodes [144]. Figure 7 sketches the transmembrane potential across (b) a symmetric model membrane formed from similar lipid leaflets and possessing a null overall potential, in comparison to (c) an asymmetric membrane where the leaflets are formed with two different lipids leading to the presence of a membrane asymmetric potential, *Δφ ≠ 0* [138,139,144,155]. Membrane potential is a key element in conducting membrane electrophysiological studies and in characterizing membrane surface interactions. It is traditionally measured by equating it to a compensating external electrical field as will be explained in Section 3.2. Consequently, electrophysiological techniques readily allow for measurements of membrane asymmetry or the membrane transverse structure, while measurements of lateral variations within the membrane are more challenging.

From a surface chemistry point of view, the resulting model membrane is a thin film separating two fluid-fluid or fluid-solid mediums and thus possesses a surface tension, *γ_b_*, governed by a balance of attractive and repulsive forces, and expressed as excess energy per unit area [156]. The membrane tension indicates the favorability of this surface in the system and mainly depends on the phospholipids-solvent combination used [136,139], but can also be altered electrically [137,157] or mechanically [158]. Membrane surface tension allows for the calculation of the total energy of the system leading to membrane mechanics understandings otherwise unexplained [136]. The following sections present the most adopted electrophysiological techniques aiming at investigating one or a combination of these four characteristics, while highlighting the connection between model membrane setup and the electrical approach and interpreting the results into membrane findings.

### 3.1. Conductance Measurements 

**Figure 8:**
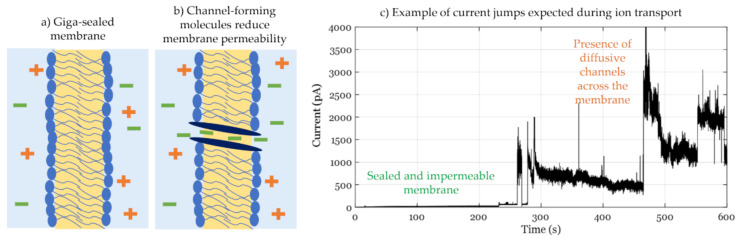
Membrane conductance studies. (**a**) Giga-sealed membranes present tightly packed lipid sheets where the hydrophobic layer inhibits ionic transport (**b**) Once a channel forming molecule–peptides, polymers, or others–integrates across the membrane leaflets, ions transporting channels are created and the gross membrane conductance is increased. This can be detected by applying a constant DC voltage and monitoring the membrane-generated current. (**c**) Adopted from “Makhoul-Mansour, M.M., et al., Photopolymerized microdomains in both lipid leaflets establish diffusive transport pathways across biomimetic membranes. Soft matter, 2019. 15(43): p. 8718-8727”. Example of current behavior upon increase in membrane conductance. Conductance here is induced by the presence of channel-forming photopolymerizable phospholipids in DIBs generating defects in the membrane.

Conductive channels are the cell’s primary mode of exchange across the near-impermeable double layer [1]. These are either formed naturally by the cell or synthetically by the interference of foreign agents such as the case of an actively attacked bacterial cell wall [159,160]. Depending on the cell’s type, cycle, and surrounding, these molecules form different configurations of pores or defects across the lipid barrier, detected as an increase in the membrane conductance [150]. Model membranes present a reliable platform to estimate the disruption of these agents at the cellular wall. Broadly, a conductance study relies on tracking the current’s offset while applying a DC voltage. The application of a constant DC voltage without an alternating component minimizes the capacitive currents and focuses solely on the resistive portion. Jumps in the recorded current and deviations from the reference level indicate the temporary disruption of the near-impermeable lipid barrier. Eliminating the capacitive currents from Equation (1), the resistive current is expressed as shown in Equation (2):

(2) $$I_{R}=G_{m}V_{DC\;}$$

where *V_DC_* is the DC voltage applied and *G_m_* is the membrane conductance. In these studies, the membrane’s innate or base resistance must be controlled for successful experiments and reliable data. In fact, the membrane resistance must be in the giga-ohms range, conventionally called a giga-sealed membrane, as illustrated in Figure 8a, prohibiting any ion transport that is not induced by the biomolecule in question and thus enabling single channel recordings, as seen in Figure 8b. 

Conductance measurements reveal the mechanics of channel or pore-forming molecules, characterizing their dependence on concentration [161], membrane surface charge [162], and applied electrical field [163]. In addition, these measurements track the behavior of selective channels while varying the ionic species and their concentrations mimicking the ionic selectivity quality of biological membranes [164]. Conductance measurements require giga-sealed membranes to clearly observe agent-induced conductance events. Wu et al. presented a thorough study on the interaction of a variety of peptides in pore-spanning membranes, investigating if the cell membrane is their primary target when attacking bacterial walls [21]. To mimic the surface charge of gram-negative bacteria, they appropriately mixed zwitterionic and anionic phospholipids. They noticed that only a negative voltage allowed for conductive channel formation, which was explained by the fact that the peptides in questions were cationic demanding a negative surface charge for surface adhesion highlighting membrane electrostatics. Also using pore-spanning membranes, Ashrafuzzaman et al. investigated the effect of gramicidin-S at the bacterial membrane [165]. They altered with the membrane surface charge and permeability by testing the peptide with zwitterionic phospholipids, then mixed with 20% anionic phospholipids, with and without the addition of cholesterol as the latter reduces membrane permeability [50,166,167]. Results showed that anionic and cholesterol-free membranes showed the highest interaction–higher conductance for a longer time–than neutral rigid membranes. 

The DIB platform has also been used for conductance measurements, enabling flexible formation of lipid mixtures through control of lipid-dispersed droplets and solvent solutions. This allows for alternating the membrane’s rigidity [166] and surface charge [151], in addition to the easy formation of asymmetric membranes [167], all affecting membrane-surface interactions. The use of DIBs in conductance studies has revealed multiple membrane mechanics including the activities of proteins, nanoparticles, and even the phospholipids themselves. The DIB platform enables mechanical membrane tension manipulation through parallel displacement of one droplet with respect to the other, allowing for the study of mechanosensitive protein channels [68,158,168]. de Planque et al. used the DIB platform to further investigate the effect of silica nanospheres on protein-free membranes [169]. The lipids were dispersed in the oil phase, whereas the nanoparticles were dissolved in one of the droplets indicating the trans side. The immiscibility of the liquids acts as a physical separation between the lipids and the silica particles inhibiting any pre-membrane interactions that might alter the resulting structure. Membrane conductance was tracked for various nanoparticle concentrations quantifying their effect on membrane structure as well as their toxicity level. The DIB platform does not limit testing channels formed through external peptides but also through defects between the phospholipids themselves. Punnamaraju et al. demonstrated the behavior of 23:2 DiynePC photopolymerized phospholipids before and after UV light curing [170]. It was shown that these phospholipids form diffusive channels across the membrane only when they have been polymerized under UV light. Building on these original findings, Makhoul-Mansour et al. showed that additionally, pores only form when these phospholipids are present in both leaflets, example shown in Figure 8c [155]. In this case, the DIB platform allowed the comparison between symmetric and asymmetric membranes for lipids-in-water and lipids-in-oil scenarios.

### 3.2. Electrowetting and Electrocompression-Based Techniques

The previous method focused on tracking the membrane conductance under a direct voltage, *V_DC_*. The techniques presented herein shift the focus from membrane conductance to membrane capacitance, which is present in response to an alternating voltage. In this section, it will be assumed that the membranes are always giga-sealed, meaning there is no leak or permeability across the bilayer. The resistive current will be attenuated and thus ignored, only the capacitive current will be considered. 

#### 3.2.1. Dynamic Membrane Capacitance in Response to an Electric Field

**Figure 9:**
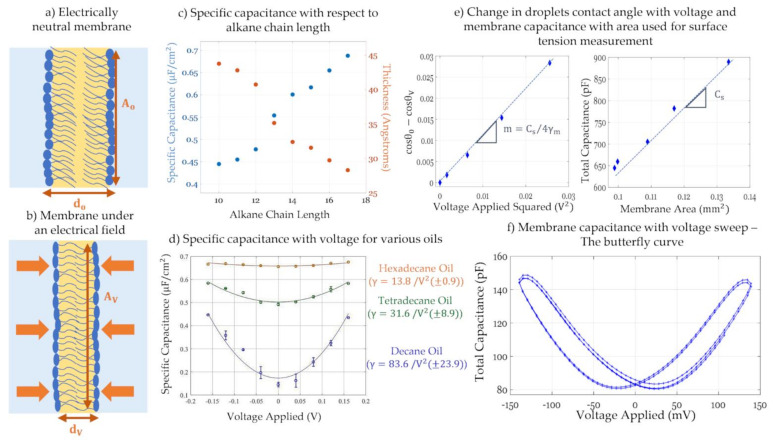
(**a**) With zero intramembrane electric field, the membrane is at its relaxed state with an initial geometry suitable for the system’s equilibrium. (**b**) When an electrical field is applied, the membrane undergoes electrocompression-reduction in thickness due to attractive coulomb forces and if the setup allows, electrowetting-increase in membrane area due to a reduction in surface tension. (**c**) Reproduced from “Gross, L.C., et al., Determining membrane capacitance by dynamic control of droplet interface bilayer area. Langmuir, 2011. 27(23): p. 14335-42.” Specific capacitance and thickness of membranes composed of the same phospholipids, but with varying alkane chain length shows the solvent effect on membrane properties. (**d**) Reproduced from “El-Beyrouthy, J., et al., A new approach for investigating the response of lipid membranes to electrocompression by coupling droplet mechanics and membrane biophysics. Journal of the Royal Society Interface, 2019. 16(161): p. 20190652”. Specific capacitance with respect to voltage for different solvents showing the latter’s effect on the membrane’s elasticity. (**e**) Adapted from “El-Beyrouthy, J., et al., A new approach for investigating the response of lipid membranes to electrocompression by coupling droplet mechanics and membrane biophysics. Journal of the Royal Society Interface, 2019. 16(161): p. 20190652”. The DIB set up allows for a direct in-situ measurement of monolayer surface tension by monitoring the change in droplets contact angle, and thus membrane tension, with voltage, assuming constant membrane thickness. (**f**) Changes in membrane capacitance due to a relatively fast voltage sweep leads to a butterfly shaped curve. The point of overlap indicates transmembrane potential compensation.

Due to their fluidic nature, model membranes are soft capacitors able to react and thin to new dimensions in the presence of externally applied forces. Under an electric field, a lipid bilayer undergoes two main phenomena: a reduction in its surface tension leading to lateral expansion: electrowetting; and thinning between its leaflets: electrocompression; as seen in Figure 9a,b. Similar to a sessile droplet sitting on a semi-conductive surface, electrowetting is the reduction in the membrane tension under an electric field [157,171,172,173]. Reducing the membrane surface tension enhances its favorability in the system leading to a relaxation or expansion in its area. This phenomena is recently used as the driving force for multiple droplet on a microchip manipulations [174,175] as well as pore gating through membrane tension alterations [173]. However, this expansion is not always possible given boundary conditions and constraints on the model membrane. In pore-spanning membranes for example, membrane area is bounded by the size of the orifice leading to minimized adjustments. Whereas, in droplet-based techniques, the membrane is free to expand reaching the minimum energy desired under the new equilibrium, barring constraints provided by the attached electrodes. Simultaneously, dielectric stress leads to attractive coulomb forces causing the leaflets to thin in the transverse direction [73,136]. This is denoted as electrocompression which occurs in all model membranes and whose magnitude depends primarily on the selected solvent and slightly on the lipids used. Combining the two phenomena, the introduction of an electric field across the membrane leads to membrane thinning and expansion when possible which causes a change in the membrane total capacitance in response to voltage, or electrostriction. The geometrical dependence of the membrane capacitance is explained by the approximation of planar membranes as parallel plate capacitors:

(3) $$C_{m}=C_{s}A_{m}=\frac{\varepsilon }{d}A_{m}$$

where *C_s_* is the membrane specific capacitance or capacitance per unit area, *C_m_* is the membrane total capacitance, *A_m_* is the membrane area, *ε* is the effective permittivity considering that of the hydrocarbon chains and *d* is the membrane dielectric thickness. Thus, an increase in membrane area and a reduction in its thickness cause an overall increase in the total capacitance, which is quadratic with respect to the voltage, or linear with the voltage squared [145], as seen in Figure 9d. Considering Equation (1), membrane generated capacitive current depends not only on the alternating voltage but also on the consequential variation in membrane capacitance, *C*(*V*). Membrane generated current and changes in capacitance with voltage follow these equations:

(4) $$I\left(t\right)=C\left(V\right)\frac{dV}{dt}+V\left(t\right)\frac{dC}{dt}$$

(5) $$C\left(V\right)=C_{0}\left(1+\alpha {\left(V+∆\varphi \right)}^{2}\right)$$

where *Δφ* is the membrane asymmetric potential, *C_0_* is the minimum membrane capacitance corresponding to zero total electric field, and *α* is the electroresponse coefficient. The value for *α* varies with frequency and should not be confused with the steady state response to a voltage denoted γ, in Figure 9d. In the following paragraphs, electrowetting and electrocompression techniques based on tracking changes in membrane capacitance with respect to the electrical field will be discussed as these unfold multiple membrane properties such as membrane composition, monolayer surface tension, membrane potential, and others. It is assumed that the alternating component of the voltage signal does not influence the membrane dimensions.

The general approach for tracking capacitance with changing voltage is by applying a signal of the form: $V\left(t\right)=V_{DC}+V_{0}sin\left(\omega t\right)$ across the membrane. Varying the value of *V_DC_* in a slow step function, the equilibrium capacitance is calculated at each step. Total capacitance amplitude is obtained by measuring the output current amplitude and using the capacitor voltage-current relation, as such: $C_{V}=I/\left(\frac{dV}{dt}\right)$. The change in capacitance with time in this case is set to zero, dC/dt≈0, as the equilibrium value is of interest. As for the membrane area, it is mainly obtained via visual estimations including light microscopy. Combining the capacitance with the membrane area, the specific capacitance and thus the dielectric thickness can also be calculated, based on Equation (3). Furthermore, plotting the equilibrium capacitance with respect to *V_DC_*, leads a quadratic equation as seen in Equation (5) and in Figure 9d. The resulting parabola is centered at zero in the case of symmetric membranes, but in the case of asymmetry, it is shifted by a compensating voltage that equals in magnitude but opposites in field direction to the membrane asymmetric potential, i.e., $∆\varphi =-V_{DC}$. At this voltage, the membrane initial electric field is compensated. This is denoted as the minimum capacitance technique to obtain the membrane potential [138,145]. 

In this process, one must be mindful of the signal frequency as well as the equilibration time between each voltage step. In fact, membrane impedance, as shown in Figure 7a, is frequency dependent, so the frequency adopted, *ω*, must fall within a certain range where the capacitance dominates over the resistance [176,177]. Even though the model membrane is impermeable, and the conductance is theoretically zero, if using an inappropriate frequency, the generated current may include a resistive component while considered as purely capacitive. Second, the wait time between the voltage steps must allow for the membrane to reach its new equilibrium, so that the capacitance indicates the steady state value. Mainly depending on the oil’s viscosity [72,126,178] but also on the membrane size, the time needed for the membrane to reach steady state differs from one bilayer to another and must be adjusted accordingly.

Emulsion-based membranes present a suitable platform in electrowetting and electrocompression analysis as their fluidic nature enables an unconstrained response to the electrical field and a direct connection between droplets geometry and membrane electrophysiology [179]. The following paragraphs focus on some of the recent innovations in membrane characterization developed through droplet-based membranes. Gross et al., adopted the DHB platform to track membrane capacitance with alternating areas via changes in voltage [73]. In that work, multiple model membranes were formed with the same phospholipids while varying the organic solvent, from short to long chain length oils. The DHB platform allows for a direct visualization of the membrane area through an inverted microscope, enabling accurate thickness calculations. Findings included a reduction in membrane thickness and elasticity as the oil chain length increases, as observed in Figure 9c. In fact, low chain alkane leaves residuals in between the leaflets during monolayers adhesion, leading to a solvent-full membrane able to significantly thin by expelling these residuals. Higher chain oils will not remain between the leaflets leading to a solvent-free membrane with not much room for further thinning. In this work the contact angle was estimated assuming a spherical cap geometry of the droplet. 

Direct measurements of this contact angle may be achieved visually in the DIB approach. Taylor et al. presented a DIB based approach that relies on an altered Berge-Lippmann-Young equation to calculate the monolayer surface tension in-situ [137]. In the DIB setup, the membrane tension is balanced by the two monolayer surface tensions as follows:

(6) $$\gamma_{b}=2\gamma_{m}cos\theta_{m}$$

where *γ_b_* and *γ_m_* are the bilayer and monolayer tensions, respectively, and *θ_m_* is half the contact angle in between the droplets. Through an inverted microscope, direct contact angle measurement is possible and hence the calculation of the bilayer tension according to Equation (6), providing that the monolayer surface tension is separately measured, using for example the pendant drop approach [142,180]. Alternatively, Taylor et al. proposed balancing the energy of the applied electric field with the reduction in membrane tension, and assuming negligible electrocompression constant membrane thickness the monolayer tension was calculated. Berge-Lipmann-Young equation provides the balance of forces between the electric stress and the reduction in membrane tension: $∆\gamma_{b}=E_{elec}.\;$Using Equation (6) and the equation of a charged capacitor, the Berge-Lipman-Young equation specific for DIBs was introduced [137]:

(7) $$cos\theta_{0}-cos\theta_{V}=\frac{C_{s}}{4\gamma_{m}}V^{2}$$

The slope of this equation as well as the membrane specific capacitance are obtained graphically as seen in Figure 9e, leaving the monolayer tension as the only unknown in Equation (7). Monolayer surface tensions obtained from this equation were compared to the ones form the pendant drop technique validating the accuracy of the approach. Building on these two innovations, El-Beyrouthy et al. presented an enhanced DIB setup where the droplets are visualized from the bottom and the side view, allowing for simultaneous contact angle and membrane area measurements revealing additional membrane properties under dielectric forces [136]. This alteration allows for the direct measurement of a varying membrane specific capacitance, or thickness, considering electrocompression of the leaflets. In a similar effort, Rofeh et al. adjoined a side-view camera on the DIB platform allowing in-situ measurement of the monolayer tension through the pendant drop algorithm [143]. 

Capturing the change in membrane capacitance with a varying electric field was also investigated by Schoch et al. for the purpose of quantifying the membrane intrinsic potential and other membrane properties [144]. Schoch et al. formed solvent-full asymmetric pore-spanning membranes where membrane asymmetry was introduced through salt concentration mismatch causing surface potential difference. In that work, membrane capacitance was tracked with respect to a voltage sweep rather than voltage steps. The applied signal was composed of a high frequency low amplitude sinusoidal voltage added to a low frequency high amplitude triangular one driving the voltage sweep. The high frequency sinusoidal voltage was used for the capacitance calculations, whereas the low frequency signal alternates between *± V_DC_* within a period of $T=1/f_{slow}$. Membrane capacitance with respect to the slow voltage forms a butterfly shaped curve, as seen in Figure 9f. It has been shown that the butterfly curve is centered at the voltage compensating for the membrane asymmetric potential [144,181]. Hysteresis is observed since the membrane is not allowed sufficient time for equilibration. Hysteresis is linked to how much and how fast the membrane responds under changes in electric force, depending on the solvent and the frequency used for the voltage sweep [181]. The use of a solvent-full membrane produces greater sensitivities to electrocompression, enabling changes in capacitance that are easily detected. Since pore-spanning membranes were used in the original work, the change in total capacitance can be directly tied to changes in thickness as the membrane area is constrained, removing this additional variable.

#### 3.2.2. Membrane Current Analysis and Attenuation Techniques

**Figure 10:**
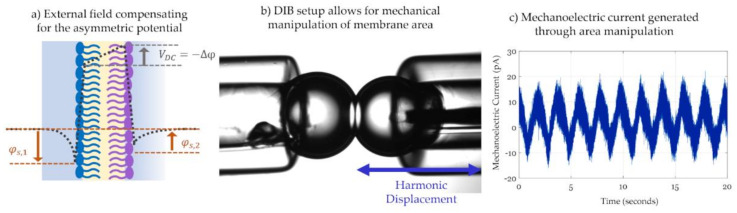
(**a**) Example of the transmembrane potential profile of a generic asymmetric membrane. Having two different leaflet compositions leads to an offset in the overall transmembrane potential, denoted as the membrane asymmetric potential, *Δφ.* The latter is composed of the difference between the surface potentials and the dipole potentials between the lipid sheets and can be measured based on the attenuation of an electrocompression-generated current. (**b**) The DIB setup allows for mechanical compression of the membrane through the displacement of one droplet with respect to the other, leading to mechanical adjustment of the membrane area. (**c**) This mechanical displacement leads to the generation of a mechanoelectric capacitive current.

The previous paragraph presented the capacitance dependency on a changing electric field and its links to membrane properties and energetics. This paragraph focuses on attenuating the current generated across the membrane to find the minimum field. Analyzing the voltage necessary for minimum current instead of capacitance trends with respect to voltage enables faster and more frequent measurements. However, this comes at the cost of requiring an approach for attenuation. In 1980, Sokolov et al. first presented the Intramembrane Field Compensation (IFC) technique for measuring membrane asymmetric potential based on solvent-full pore-spanning membranes [182]. Lipid composition mismatch between the leaflets causes a membrane potential offset, as described in Figure 10a. The membrane asymmetric potential necessitates the application of an opposing external electrical field to compensate for its influence on membrane dimensions, and its measurement allows for investigating complex membrane biophysics [183] including membrane-particle interactions [184,185].

IFC exploits the rapid changes in the membrane’s thickness with an oscillating electrical field. To summarize, a voltage signal of the following form is applied: $V\left(t\right)=V_{DC}+V_{0}\cos\left(\omega t\right)$. The voltage signal has a direct component, *V_DC_*, and a sinusoidal component of a relatively high amplitude, *V_o,_* and high frequency, *ω.* Recalling Equation (4), membrane generated capacitive current includes changes in voltage and capacitance with respect to time. Since the instantaneous change in membrane capacitance is the driving element of this technique, it is crucial to consider both these terms of the capacitive current. The fast Fourier transform, or FFT, is then applied to the current dividing it into its harmonics. Replacing the fast change in capacitance, by: $C\left(t\right)=C_{0}\left(1+\alpha V{\left(t\right)}^{2}\right)$, and the true voltage drop across the membrane, by: $V\left(t\right)=\left(V_{DC}+\Delta \varphi \right)+V_{0}\;sin\left(\omega t\right)$, where *Δφ* is the membrane potential due to its asymmetry, into Equation (4), the first two current harmonics are as follows [186]:

(8.a) $$I_{\omega }=V_{0}\omega C_{0}\left[1+3\alpha {\left(V_{DC}+\Delta \varphi \right)}^{2}+\frac{\alpha V_{0}^{2}}{2}\right]sin\left(\omega t\right)$$

(8.b) $$I_{2\omega }=3\alpha V_{0}^{2}\omega C_{0}\left(V_{DC}+\Delta \varphi \right)sin\left(2\omega t\right)$$

As would be expected, the first harmonic, shown in Equation (8.a), is the most dominant as *ωV_0_C_0_* is a direct integration of the voltage according to the impedance of a capacitor. However, the second harmonic is the one of most interest in this technique. Equation (8.b) shows that the amplitude of the second harmonic is approximately linear with the total electrical field, $\left(V_{DC}+\Delta \varphi \right)$, and it equals zero when the applied voltage matches the membrane potential, i.e., $V_{DC}=-\Delta \varphi$, rending the oscillating voltage symmetric about a compensated intramembrane field. Thus, the technique proposed by this original work analyzes the membrane current, examines the harmonics of the signal, then alternates the DC voltage while monitoring the second harmonic until the second harmonic is attenuated. Upon attenuation, the DC voltage successfully compensates the membrane asymmetric potential. 

For the measurement of the membrane potential, IFC does not require any capacitance or even current calculations, requesting solely the attenuation of the second harmonic to its feasible minimum. Furthermore, it untangles the experimental design from any geometrical constraints such as a fixed or a variable membrane surface area or tracking the changes in membrane thickness, emphasizing its advantage over the minimum capacitance technique in measuring the membrane potential. However, the second harmonic presents a relatively small amplitude, so experimental amplifications are needed to clearly differentiate it and to have an intensified change with varying *V_DC_*. The electroresponse coefficient, *α*, describes the capacitance change with respect to voltage, as seen in Equation (5), quantifying the membrane response intensity. The original work of Sokolov et al. used pore-spanning membranes, which are typically formed with shorter chain alkanes leading to a pronounced thinning in response to the electrical field, amplifying the value of *I*_2*ω*_ and making it more susceptible to changes in *V_DC_*. Additionally, in the case of pore-spanning membranes, electrocompression is the main response to an electrical field as the membrane area is bounded by the orifice surface, making electrowetting phenomena negligible. Thus, *α* represents the electrocompression intensity of the thickness-alternating membrane. If using membranes that are not laterally constrained instead, such as DIBs, *α* would represent the total electroresponse including electrowetting and electrocompression combined and differentiating between the two phenomena requires additional calculations and considerations which would not affect the IFC design but might be used to reveal additional membrane mechanics beyond the intrinsic potential [181]. Furthermore, the alternating voltage amplitude and frequency must be tuned as well. In theory, the highest amplitude and frequency that can be experimentally provided are desired as these amplify the second harmonic amplitude. However, these values must be chosen carefully to avoid overcompensation leading to opposite results. In fact, using an overly high frequency might be too fast for the membrane to follow and thin due to the solvent viscous effects [181]. In addition, the frequency must present a capacitance-dominant impedance without interference from the electrolyte resistance [176]. Thus, the sinusoidal voltage amplitude and frequency must be large enough to amplify the membrane response, but care must be taken not to overcompensate and lead to a nonresponsive membrane or to a resistance-dominated impedance.

As explained in the previous paragraph, IFC is most effective when used on solvent-rich highly elastic membranes. This primarily includes pore-spanning membranes that multiple researchers adopted and, following the original work, used the IFC method to investigate not only membrane asymmetric potential but additional membrane properties and mechanics [184]. Pohl et al. used solvent-full BLMs and applied IFC to track pH-driven lipid flipflop events [183]. Advancing the approach, Passechnik accounted for the heterogeneity of the membrane layers and re-developed the current harmonic equations while considering the electric stress and membrane compression moduli [187], allowing for the localization of charges across the double layer levels [185]. Solvent-full droplet-based membranes are also favorable for IFC measurements. In fact, El-Beyrouthy et.al built highly elastic membranes by forming DIBs with decane oil [73], and combining this membrane with an automated control system, the IFC fundamentals were successfully utilized for obtaining a rapid and real-time reading of asymmetric membrane potentials [188]. 

Inspired by the alternating change in membrane capacitance, Freeman et al. used the DIB platform to create a droplet compression system that generates mechanoelectric current [189]. Figure 10b shows how the droplets-based platform is manipulated to generate current through displacement rather than the application of an electric field. Equation (4) shows that the capacitive current across the membrane is enabled through two components: an alternating voltage and an alternating capacitance. The initiative of this work focuses on the alternating capacitance (*dC*/*dt*) achieved by varying membrane area through compression of the droplet pair in a rhythmic fashion using a piezoelectric actuator [158,189]. An example of this mechanically induced current is shown in Figure 10c. The ability to mechanically compress the membrane is possible through the fluidic nature of DIBs and the change in area was shown to be substantial enough to be detected by tension-driven peptides [158,190]. However, the main restriction of this technique remains in the maximum change in area possible by the interface. Similarly to the frequency issue discussed in the IFC technique, the displacement frequency has to be high enough to increase the current amplitude but slow enough to allow the membrane to respond accordingly, otherwise the current is attenuated. Additional mechanoelectric work showed that a lower displacement frequency promotes a higher change in membrane area [158]. 

### 3.3. Electroimpedance Spectroscopy 

**Figure 11:**
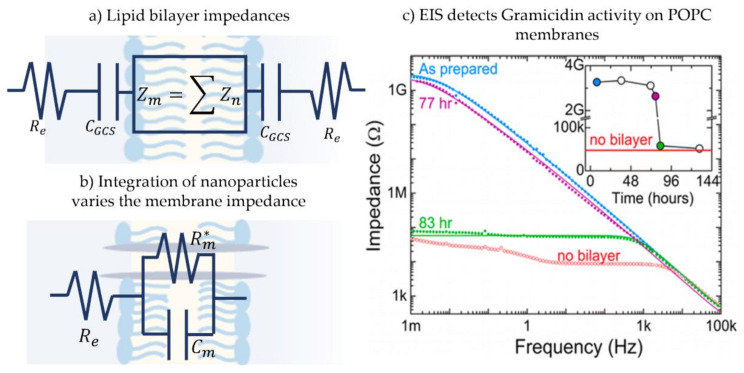
(**a**) The overall membrane electrical behavior is often more complex than a resistor and a capacitor in parallel. The electrolytes resistance *Re* and the capacitance of the electrical double layer *C_GCS_* are also present in the electric circuit, but the membrane impedance typically dominates the response. However, the double layer itself and its surrounding can be divided into tuned impedance layers depending on the hypothesis in question and each layer is detected through varying frequencies. (**b**) A common electrical circuit when investigating channel-forming proteins in membranes: the electrolyte solution resistance, *R_e_*, in series with membrane capacitance, *C_m_*, and membrane resistance, *R_m_*. The latter is variable in the presence of membrane-protein activities. (**c**) Copied from “Korman, C. E., et al. (2013). “Nanopore-spanning lipid bilayers on silicon nitride membranes that seal and selectively transport ions.”29(14): 4421-4425. Membrane conductance was tracked via EIS investigating the effect of Gramicidin on POPC membranes.

The simple electrical representation of the membrane as a capacitor in parallel with a resistor is true under the condition that the frequency applied leads to a dominant membrane impedance, $Z_{m}\left(\omega \right)=1/\left(G_{m}+j\omega C_{m}\right)$, where *G_m_* and *C_m_* are the membrane specific conductance and capacitance, respectively [191]. This impedance is that of the membrane core, however, the entire fluidic double layer structure contains additional regions of interest. In addition to the core membrane impedance, the electrical double layer capacitance, *C_GCS_*, and the electrolyte solution resistance, *R_e_*, are present when considering the entire electrical circuit [117], as illustrated in Figure 11a. Generally, and for the ease of analysis, the impedance of the electrical double layer at the hydrophilic-aqueous interface, *C_GCS_*, is either ignored or added to the membrane impedance. The reasoning behind this is that the capacitance of this layer is significantly high compared to that of the membrane, leading to a much smaller influence on the system’s equivalent impedance. While commonly being an unwanted impedance, the electrolyte resistance is avoided by using an appropriate frequency range [176] and a specific salt concentration [192].

Electroimpedance spectroscopy, or EIS, is a frequency-based analysis that considers the model membrane’s total impedance response with respect to a frequency sweep: Bode or Nyquist plots [191,193]. It is distinguished from previously discussed techniques in the fact that it does not investigate changes in the intramembrane field but focuses solely on its electroimpedance response. In this analysis, each model membrane layer, or membrane component, is considered as a separate impedance element composed of a real and imaginary part indicating its conductance and capacitance, respectively. EIS consists of sending a small amplitude alternating voltage across the membrane while performing a frequency sweep. The voltage amplitude must be small enough to avoid any nonlinear effects related to the presence of a high electric field [194]. As for the frequency range, it generally ranges from a few *mHz* to several *kHz,* depending on the resolution of the impedance analyzer [191,195]. In addition, the data sampling frequency and the number of samples must be adjusted during the sweep to obtain evenly distributed data throughout the frequency range. The generated current and applied voltage are used to get the total impedance response impedance amplitude and phase angle. The generated Bode plots are then compared to the modeled equivalent circuit leading to capacitance and conductance measurements corresponding to various membrane layers. The electrical model for the membrane may be altered as needed dependent on the experimental data, revealing additional membrane layers properties such as area defects [117,196].

Electroimpedance spectroscopy is commonly utilized on solid supported membranes, as this specific setup allows for the direct connection between bilayer and electrode, removing additional undesired impedance layers and increasing the frequency range otherwise limited. Figure 11b presents a common electric circuit used to describe these membranes under EIS. Note that the membrane resistance, *R_m_,* is variable in the presence of a channel-forming biomolecule and tracking membrane equivalent impedance mirrors changes in the conductance highlighting membrane-biomolecule interactions. In solid supported membranes, EIS helps detecting membrane formation [118,197], separates the multi-layers of this membrane [98,198,199], as well as detects and localizes biomolecule attachment [99,191,195,200,201,202]. Stelzler et al. utilized EIS on solid supported membranes made through two different approaches–LB/LS and vesicle fusion and compared the mechanics of ligand bindings in these membranes [117]. In EIS analysis, the electric circuit is adjustable as the electrical components can be either divided into sub-impedances or grouped together. For example, Karolis et al. investigated the effect of cholesterol on egg lecithin bilayers while being interested in the specific location this sterol made the greatest effect on the phospholipids [203]. To do so, the electric circuit adopted consisted of 4 impedances each represent a different part of the phospholipid molecule: acyl chain, carbonyl, glycerol bridge and phosphatidylcholine. Whereas, Romer and Steiner used the EIS technique to obtain electrical properties of a newly developed model membrane, a hybrid between pore-spanning membrane and solid supported membrane [194]. For their hypothesis, the membrane was considered as one impedance since the interest was on the membrane as one entity with no need for added complexity. Figure 11c. shows the work of Korman et.al, who utilized EIS spectra to measure multiple POPC nano-membranes equivalent impedance [204]. This work characterized these membranes and investigated the effect of gramicidin, showing how this channel-forming protein increases the membrane conductance, and how the influence of gramicidin can be reduced with divalent cations, such as when CaCl_2_ is used in the buffer solution. More recently, EIS has been utilized to characterize microcavity pore-suspended lipid bilayers for detecting membrane-drug activity [205], as well to investigate the adsorption and attachment of lipid vesicles on a solid substrate [206]. Not limited to solid-supported membranes, EIS has been used on networks of membranes formed by adhesive emulsion systems such as a network of DIBs, where the impedance response allows for multiple membrane studies and total network analysis [176,177]. 

## 4. Summary of Methods for Formation and Characterization

This review presents a collection of common model membranes developed to mimic the structure of cellular membranes in a controlled environment. Section 2.1. discusses the formation of liposomes, or lipid vesicles, which are similar in shape and size to natural membranes. Section 2.2. presents pore-spanning membranes formed at a hydrophobic orifice, whose high membrane resistance and elasticity allowed for the development of various electrophysiological approaches. Section 2.3. discusses solid supported membranes, which are mechanically robust and highly used in membrane-protein studies. Section 2.4. includes two droplet-based membranes, which allow for direct correlation between emulsion mechanics and membrane biophysics. Table 1 summarizes these model membrane manufactures and their resulting properties. 

Table 2 summarizes the second part of this review, which focuses on three fundamental electrophysiological approaches as well as their ongoing development. In Section 3.1, the membrane conductance is tracked to analyze membrane structure and surface interactions. In Section 3.2, dynamic membrane capacitance and alternating current are utilized for revealing several membrane properties such as thickness, elasticity, surface tension and asymmetric potential. In Section 3.3. electroimpedance spectroscopy allows for the tuned and detailed study of lipid membranes sub-layers. 

## 5. Conclusions

Cellular membranes are complex structures that facilitate a variety of intertwined functions in living organisms. Due to their complexity, it is often infeasible to untangle the variables responsible for their physiological properties and interactions. Therefore, synthetic model membranes are routinely used, mimicking the cell membrane’s simple structure while presenting a flexible and tunable platform for the isolation and study of specific membrane biomechanics. These artificial membranes differ from the naturally occurring membranes as a result of their selected mode of assembly. The default impermeability of most model membranes makes them highly sensitive to minute changes in conductance, leading to accurate conductive channels’ recordings for in-depth membrane-nanoparticles investigations. Furthermore, and depending on the solvent used, model membranes possess an enhanced membrane elasticity making them highly responsive to electrical forces. This amplified soft response allows for membrane structure investigations, bending stiffness studies and for the direct measurement of membrane asymmetric potential. Free-standing model membranes such as droplet-based membranes result in a unique link between droplets geometry and membrane biophysics including membrane tension and membrane electrostatics.

In this review we examined several common methods for producing these model membranes in the laboratory, highlighting differences in the produced membranes. Next, we discussed how these differences may be exploited for enabling alternative techniques for characterizing the membrane properties, focusing in particular on membrane-particle interactions. Model membranes are a simple representation of natural membranes and despite their undeniable deviation in shape and innate characteristics from biological membranes, they allow for investigations that might be more challenging in natural systems. 

## Figures and Tables

**Table 1:** Summary of the discussed model membrane manufactures.

**Model** **Membrane**	**2.1. Liposomes**	**2.2. Pore-Spanning** **Membranes**	**2.3. Solid Supported Membranes**	**2.4. Emulsion-Based Membranes**	
				**2.4.1. Droplet on Hydrogel Bilayers**	**2.4.2. Droplet Interface Bilayers**
**Description**	Lipid vesicles formed in an aqueous environment	Lipid bilayer formed at the orifice of a solid separator between two aqueous baths	Lipid bilayer formed on a solid support submerged in an aqueous solution	Lipid bilayer formed at a droplet-hydrogel interface	Lipid bilayer formed at a droplet-droplet interface
**Manufacturing Techniques**	Electroformation Phase Transfer Microfluidic Jets	Solvent painting Monolayers folding	Langmuir-Blodgett/ Langmuir-Schaefer Vesicle fusion	Microfluidic droplet deposition and manipulation in oil reservoirs	
**Advantages**	Similar in geometry and dimensions to natural membranes	Well-packed, high impedance membranes Isolate transverse properties	Mechanically robust, stable, and long-lasting membranes	Full membrane area visualization	Direct tension measurements Simple formation of asymmetric membranes

**Table 2:** Summary of the discussed electrophysiology-based techniques.

**Electrophysiology** **Technique**	**3.1. Conductance Measurements**	**3.2. Electrowetting and Electrocompression**		**3.3. Electroimpedance Spectroscopy**
		**3.2.1. Dynamic Capacitance**	**3.2.2. Current Attenuation**	
**Fundamental Equation**	$I=G_{m}V_{DC\;}$	$I\left(t\right)=C\frac{dV\left(t\right)}{dt}$	$I\left(t\right)=\left(V_{DC}+∆\varphi \right)\frac{dC\left(t\right)}{dt}$	$Z_{m}\left(\omega \right)=1/\left(G_{m}+j\omega C_{m}\right)$
**Experimental** **Approach**	Applying constant DC voltage and tracking the current	Applying step-DC voltage and calculating the equilibrium capacitance	Attenuating the current harmonics through varying the applied voltage.	Generating Bode or Nyquist plots and comparing them to the expected model circuit
**Common** **Applications**	Measuring channel-forming mechanisms of disruptive agents	Calculating membrane potential, dielectric thickness, and monolayer surface tension	Measuring membrane potential and rigidity Detecting intramembrane dynamics	Detection and localization of molecular adsorption and sensor platforms
**Experimental Requirements**	High base membrane resistance	Sufficient equilibrium time between voltage steps	Highly compressible membrane for enhancing measurements	High signal frequencies and compatible equipment.

## Data Availability

The study did not report any data.
